# Pan-cancer Multi-omics Analysis Reveals HMGN1 as a Potential Prognostic and Immune Infiltration-associated Biomarker

**DOI:** 10.2174/0109298673268718231122103638

**Published:** 2024-01-08

**Authors:** Yangyang Guo, Rongrong Zhang, Hongjie Xu, Kai Hong, Kenan Cen, Yifeng Mai, Zhixuan Wu

**Affiliations:** 1 Department of Thyroid and Breast Surgery, The First Affiliated Hospital of Ningbo University, Ningbo, 315010, Zhejiang, People's Republic of China;; 2 Department of Pathology, The First Affiliated Hospital of Wenzhou Medical University, Wenzhou, 325000, Zhejiang, People's Republic of China

**Keywords:** HMGN1, pan-cancer, multi-omics, prognosis, immune infiltration, tumor microenvironment

## Abstract

**Background:**

The High Mobility Group Nucleosomal Binding Domain 1 Gene (HMGN1) is crucial for epigenetic regulation. However, the specific function of HMGN1 in cancer development is unclear.

**Methods:**

Raw data on HMGN1 expression were procured from Genotype-Tissue Expression (GTEx), the University of Alabama- Birmingham CANcer data analysis Portal (UALCAN), and The Cancer Genome Atlas (TCGA). Thereafter, the pan-cancer analysis was implemented to understand the HMGN1 expression patterns, prognostic value, and immunological features. Furthermore, the Gene Set Enrichment Analysis (GSEA) was executed *via* R language. In addition, the relationship between HMGN1 and the sensitivity of antitumor drugs was also determined. Finally, real-time PCR (RT-PCR) experiments were carried out.

**Results:**

Pan-cancer analysis revealed that HMGN1 was upregulated in several solid tumors and was associated with pathological staging and poor prognosis. In addition, HMGN1 was found to be involved in regulating the tumor microenvironment. The GSEA enrichment analysis indicated that HMGN1 assisted in the regulation of oncogenic processes, especially metabolic and immune pathways. Furthermore, HMGN1 expression was linked to microsatellite instability (MSI) and tumor mutational burden (TMB) across diverse tumor types. RT-PCR assays indicated that HMGN1 was overexpressed in the gastric and breast cancer cell lines and tissues.

**Conclusion:**

This study highlighted the potential of HMGN1 as a biomarker for pan-cancer analysis.

## 1 INTRODUCTION

Cancer has emerged as a significant contributor to premature mortality among the global population, owing to both the expanding populace and the advancing age demographics [1-4]. Despite the escalating societal burden of cancer, significant advancements in cancer treatment have yet to be achieved. The development of cancer immunotherapy, as well as the advancement and establishment of public databases like the Cancer Genome Atlas (TCGA), helped in studying the pan- cancer expression profiles of specific genes to understand their relationship with the clinical prognosis and associated signaling pathways, thereby improving the discovery of novel cancer therapeutic targets [5, 6].

All vertebrates possess the essential epigenetic regulator gene, *i.e.*, High mobility group N (HMGN), which selectively binds to the nucleosomes and exerts its role through chromatin remodeling [7-11]. The High Mobility Group Nucleosomal Binding Domain 1 Gene (HMGN1) belongs to the HMGN family and is a triplet gene on chromosome 21. It facilitates chromatin disassembly by binding to the nucleosome core structure and is associated with acetylated lysine 27 on histone H3 [12, 13]. Earlier reports have stated that HMGN1 influences the differentiation of ocular, neuronal, germ, and pancreatic cells due to its essential role in embryogenesis [8]. Furthermore, numerous reports have stated that HMGN1 participates in DNA repair mechanisms [14-17]. The functional relationship between HMGN1 and carcinogenesis, including leukemia and stomach cancer, has been highlighted in recent studies [18-21]. However, the exact function of HMGN1 in various tumor types is still unknown.

The HMGN1 expression in different types of cancers and its potential use in cancer diagnosis and prognosis were investigated during this study using a variety of bioinformatics methodologies. Additionally, enrichment analyses were implemented to assess the biological roles of HMGN1 in various types of cancers. Furthermore, the probable involvement of HMGN1 in the anti-tumor immune response was also determined.

## 2 MATERIALS AND METHODS

### 2.1 Data Acquisition

The gene expression data for 31 samples that were derived from the online Genotype-Tissue Expression (GTEx) portal (https://www.gtexportal.org/home/, Broad Institute of MIT and Harvard), in addition to the transcriptome sequencing and related clinical data for 33 tumors derived from TCGA (https://portal.gdc.cancer.gov/, National Cancer Institute), were collected using UCSC Xena (https://xena.ucsc.edu/, University of California, Santa Cruz). CPTAC analysis based on the UALCAN (http://ualcan.path.uab.edu/analysis-prot.html, University of Alabama at Birmingham), was employed to examine the HMGN1 protein expression levels in colon, breast, lung adenocarcinoma, liver hepatocellular carcinoma (LIHC), glioblastoma multiforme, and ovarian cancers. (Supplementary file).

### 2.2 Survival and Prognostic Analysis

The clinical and survival phenotype data for every sample were derived from the TCGA database. The “ggpubr” and “limma” R tools were used to examine the clinical phenotype of the tumor stage for conducting clinical phenotype correlation analysis to investigate its association with HMGN1 expression. The “pROC” tool and the “ggplot2” R program were utilized for analyzing and presenting the transcriptome data derived from the TCGA and GTEx. To ascertain the prognostic value of HMGN1, the receiver operator characteristic curve (ROC) was plotted and the area under the curve (AUC) was computed. Furthermore, a comparative study was conducted between the HMGN1 expression profiles and patient prognosis with the help of four indicators: disease-free survival (DFS), disease-specific survival (DSS), overall survival (OS), and progression-free survival (PFS). The Cox analysis was initiated using “survivor” and “forestplot” R software, while the Kaplan-Meier (K-M) technique was employed for plotting the survival curves for every type of cancer.

### 2.3 Development of Nomograms

The association between the clinical indicators (such as sex, age, pT stage, and pTNM stage), HMGN1 expression levels, and OS of LIHC and adrenocortical carcinoma (ACC) patients were examined using the Univariate and multivariate regression analyses. The “rms” program in R software was used to plot nomograms. Furthermore, calibration curves were utilized to investigate the similarities between the actual survival and survival probabilities for 1-, 3-, and 5 years, predicted by the nomograms.

### 2.4 Correlation between HMGN1 Expression Levels and Tumor Immune Microenvironment (TIME)

The ESTIMATE algorithm was utilized to estimate the stromal and immune scores in malignant tissue samples, while the association between HMGN1 expression levels and the above scores was examined using the “ESTIMATE” and “limma” R packages. Furthermore, the relative scores of 22 immune cells in 33 types of cancers were determined using the CIBERSORT algorithm. The corr.test function was further employed to determine the association between the HMGN1 levels in particular tumors and the invasion levels of the immune cells in each cancer. Additionally, in this study, the correlation analysis of HMGN1 and immune-linked genes, such as the genes encoding chemokine receptors, immune checkpoints, major histocompatibility complex (MHC), chemokines, immunosuppressive proteins, and immune activation was conducted and the results were visualized using the heat maps.

### 2.5 Correlation of HMGN1 Expression Levels with Microsatellite Instability (MSI), Tumor Mutational Burden (TMB), and Immune Response

TMB refers to the overall cellular nonsynonymous mutation count in the coding region and is used as a biomarker to predict the effectiveness of immunotherapy [22]. MSI refers to the insertion or deletion of nucleotides at microsatellite sites [23]. The MSI and TMB scores were acquired from the TCGA database to understand the link between the HMGN1 levels in different cancers and the above factors using Spearman correlation analysis. RNA expression matrices and clinical data for the IMvigor210 immunotherapy dataset were acquired from the IMvigor210 database to compare the HMGN1 expression levels in the immunotherapy-responsive and non-responsive patients [24].

### 2.6 Enrichment Analysis

The Gene Set Enrichment Analysis (GSEA) was used in this study to investigate the biological function of HMGN1 in malignant tumors. The Kyoto Encyclopedia of Genes and Genomes (KEGG) gene sets were downloaded for this purpose through the GSEA portal (https://www.gsea-msigdb.org/gsea/downloads.jsp, Broad Institute, Massachusetts Institute of Technology and University of California). The association between HMGN1 expression levels and biological pathways implicated in each tumor was investigated, and the five pathways with significant negative and positive relationships were discovered.

### 2.7 Drug Sensitivity Analysis

The Genomics of Drug Sensitivity in Cancer (GDSC) website (https://www.cancerrxgene.org/, Wellcome Sanger Institute) provided the drug sensitivity information for 138 chemotherapy drugs. The “pRRophetic” program in the R software was used to predict the half-maximal inhibitory concentrations (IC_50_) for each drug, which indicates the response of every drug molecule.

### 2.8 Cell Culture

Here, the breast cancer cells (BT-549, MCF-7, BT474), normal human mammary epithelial cells (MCF-10A), gastric cancer cells (HGC-27, MGC-803), and human gastric mucosal cells (GES-1) were obtained from the Center for Excellence in Molecular Cell Science (Shanghai, China). The medium, which contained fetal bovine serum (10% v/v, FBS) and antibiotics (1% w/v penicillin/streptomycin), was utilized to cultivate the cells at 37°C.

### 2.9 Sample Collection

The gastric and breast cancer tissues were acquired from Ningbo First Hospital (Zhejiang, China). All the tissue samples were preserved at -80°C, immediately after surgery. Every participant in this study was asked to offer their written and informed consent before initiating this study.

### 2.10 Quantitative Reverse Transcription-based PCR Experiments (qRT-PCR)

The total RNA content was acquired from the cells and tumor samples with the help of the Trizol reagent (Takara, Japan). Thereafter, the total RNA content was reverse transcribed with the RT kit (Takara, Japan). The SYBR Green technique was employed to carry out the qRT-PCR experiment. The 2-ΔΔCt technique was employed to assess the relative HMGN1 expression; while GAPDH was utilized as the internal standard in all experiments.

### 2.11 Statistical Analysis

The data were analyzed statistically with the R tool (ver. 4.0.2). The findings have been shown as mean ± standard deviation (SD), and values that show a difference where *p<* 0.05 were statistically significant.

## 3 RESULTS

### 3.1 Differential Expression of HMGN1 between Normal and Cancer Tissues

**Fig. (1):**
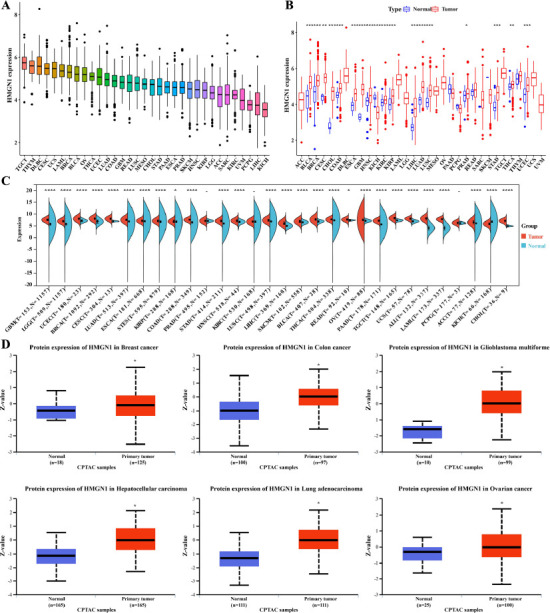
Differential expression of HMGN1 in different cancers. **(A)** HMGN1 expression in 33 types of cancer. **(B)** Comparison of HMGN1 expression between tumor and normal samples from TCGA. **(C)** The expression of HMGN1 in different cancers was analyzed using paired tumor/normal samples from TCGA and GTEx databases. **(D)** HMGN1 protein expression level in normal tissues and primary tissues of breast cancer, colon cancer, glioblastoma, hepatocellular carcinoma, lung adenocarcinoma, and ovarian cancer were analyzed using UALCAN.

Firstly, the HMGN1 expression levels in different forms of cancers were analyzed and ranked from high expression to low expression levels. The findings indicated that Kidney Chromophobe (KICH) exhibited the lowest HMGN1 expression level, while Testicular Germ Cell Tumors (TGCT) showed the highest HMGN1 expression levels (Fig. **1A**). Furthermore, the HMGN1 expression levels in the malignant and healthy samples derived from 33 forms of cancers were also compared using the TCGA dataset. Except for the tumors without appropriate normal sample data, significant variations were noted in the HMGN1 expression levels presented by the malignant and normal tissues in 18 cancers. The findings revealed that the HMGN1 gene was over-expressed in the malignant samples that were derived from different types of cancers such as Bladder Urothelial Carcinoma (BLCA), Cholangiocarcinoma (CHOL), Cervical squamous cell carcinoma and endocervical adenocarcinoma (CESC), Breast invasive carcinoma (BRCA), Glioblastoma multiforme (GBM), Colon adenocarcinoma (COAD), Head and Neck squamous cell carcinoma (HNSC), Esophageal carcinoma (ESCA), Kidney renal papillary cell carcinoma (KIRP), Kidney renal clear cell carcinoma (KIRC), Lung squamous cell carcinoma (LUSC), Lung adenocarcinoma (LUAD), LIHC, Prostate adenocarcinoma (PRAD), Thyroid carcinoma (THCA), Stomach adenocarcinoma (STAD), and Uterine Corpus Endometrial Carcinoma (UCEC), while the HMGN1 levels were downregulated in KICH malignant tissue samples, in comparison to the HMGN1 levels expressed in the healthy tissues (Fig. **1B**). In addition, all data derived from the TCGA and GTEx websites were combined and subjected to a similar data analysis using the data derived from 30 types of cancers. The findings revealed significant variations in HMGH1 expression levels in the malignant and healthy tissue samples, acquired from 27 tumors. The HMGH1 was downregulated in KICH, while it was over-expressed in 26 tumors (Fig. **1C**).

The CPTAC dataset from the National Cancer Center was then used to assess the HMGH1 protein expression levels. Fig. (**1D**) shows that, in comparison to the healthy tissues, the total HMGH1 protein expressions were elevated significantly in the malignant samples derived from the colon, breast, glioblastoma multiforme, LIHC, LUAD, and ovarian cancers. In summary, HMGN1 was strongly expressed in the majority of cancers.

### 3.2 Relationship between HMGN1 Expression and Pathological Stage

**Fig. (2):**
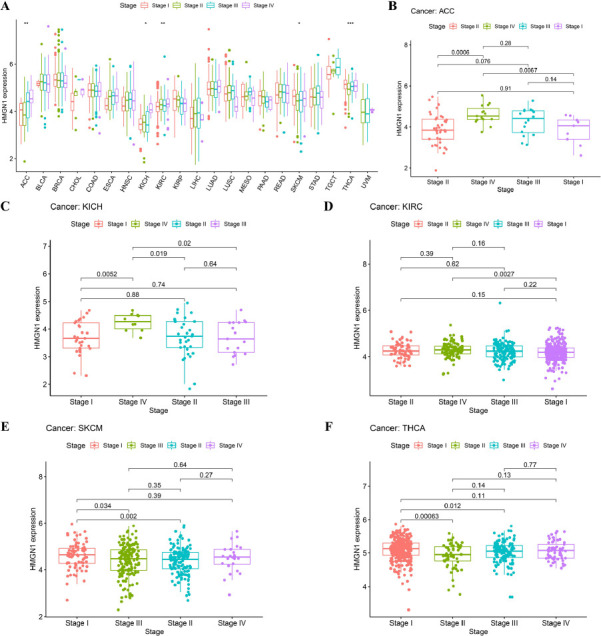
Correlation between HMGN1 expression and tumor stage. **(A)** Expression levels of HMGN1 in different stages of 21 different tumors. The expression of HMGN1 was analyzed by the main pathological stages of **(B)** Adrenocortical carcinoma (ACC), **(C)** Kidney chromophobe (KICH), **(D)** Kidney renal clear cell carcinoma (KIRC), **(E)** Skin cutaneous melanoma (SKCM), **(F)** Thyroid carcinoma (THCA).

The HMGN1 gene expression levels were also evaluated in 21 tumors at different stages and significant differences were noted in the HMGN1 expression levels in KICH, ACC, KIRC, THCA, and Skin Cutaneous Melanoma (SKCM) (Fig. **2A**). The majority of the varying HMGN1 expression levels were detected in the early (stage I) and late (stages III and IV) stages, wherein Stage IV ACC, KICH, and KIRC tumors exhibited a significant upregulation of the HMGN1 expression level compared to the stage I tumors (Fig. **2B**-**D**). On the other hand, HMGN1 expression was downregulated from stage I to stage II in SKCM and THCA, which indicated a significant difference even though a strong correlation was observed in stage I and stage III tumors (Fig. **2E**-**F**).

### 3.3 Diagnosis Value of HMGN1 in Different Cancers

In this study, the ROC curves were employed to assess the diagnostic accuracy of genetic characteristics. Different AUC cut-off values can distinguish between different diagnostic accuracies, such as high (AUC: 1.0-0.9), moderate (AUC: 0.9-0.7), and low (AUC: 0.7-0.5). Fig. (**3**) demonstrates that AUC values of the ROC analysis of this model display excellent diagnostic accuracies in CHOL, CESC, GBM, KICH, LIHC, and LUAD, while a relative diagnostic accuracy was noted in different cancers such as BRCA, BLCA, ESCA, COAD, KIRP, HNSC, LUSC, STAD, Sarcoma (SARC), and UCEC. The AUC in GBM was seen to be 1.0. In summary, HMGN1 is a highly effective tumor diagnostic marker.

### 3.4 Multifaceted Prognostic Value of HMGN1 Across Cancers

**Fig. (4):**
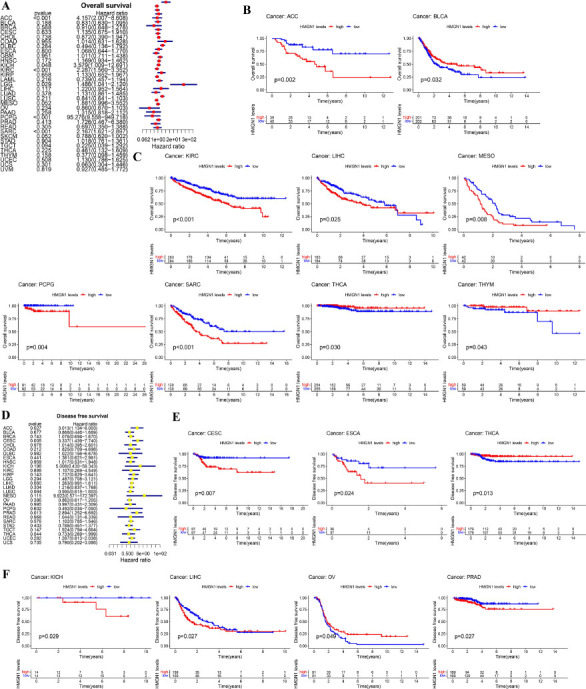
Association between HMGN1 expression and overall survival (OS) as well as disease-free survival (DFS). **(A)** Forest plot of OS associations in 33 types of tumors. **(B, C)** Kaplan-Meier curve of the association between HMGN1 expression and OS. **(D)** Forest plot of DFS associations in 28 types of tumors. **(E, F)** Kaplan-Meier curve of the association between HMGN1 expression and DFS.

The HMGN1 levels and OS of the pan-cancer patients in the TCGA database were examined using Cox proportional risk models. The findings indicated that in patients with KICH (*p*=0.048), Lower Grade Glioma (LGG) (*p*=0.029), ACC (*p<*0.001), Pheochromocytoma and Paraganglioma (PCPG) (*p<*0.001), KIRC (*p<*0.001), and SARC (*p<*0.001), high HMGH1 expression levels were linked to poor OS values, particularly in PCPG patients showing a Hazard ratio (HR) of 95.276 (Fig. **4A**). Furthermore, the results presented in the K-M curves implied that the patients showing high HMGH1 expression levels displayed short survival time in LIHC (*p*=0.025), Mesothelioma (MESO) (*p*=0.008), PCPG (*p*=0.004), ACC (*p*=0.002), KIRC (*p<*0.001), and SARC (*p<*0.001), while in the case of BLCA (*p*=0.032), THCA (*p*=0.030), and Thymoma (THYM) (*p*=0.043), a higher HMGN1 expression level was linked to a long survival duration (Figs. **4B**-**C**). Additionally, Cox analysis for DFS showed that the HMGN1 expression was inversely associated with the prognosis of the ACC (*p<*0.027), CESC (*p*=0.005), and PRAD (*p*=0.013) patients (Fig. **4D**). Additionally, K-M analysis indicated that the patients exhibiting high HMGN1 expression levels in CESC (*p*=0.007), ESCA (*p*=0.024), KICH (*p*=0.029), LIHC (*p*=0.027), and PRAD (*p*=0.027) exhibited low DFS values; however, the opposite results were noted in the case of THCA (*p*=0.013) and OV (*p*=0.049) patients (Figs. **4E**-**F**).

**Fig. (5):**
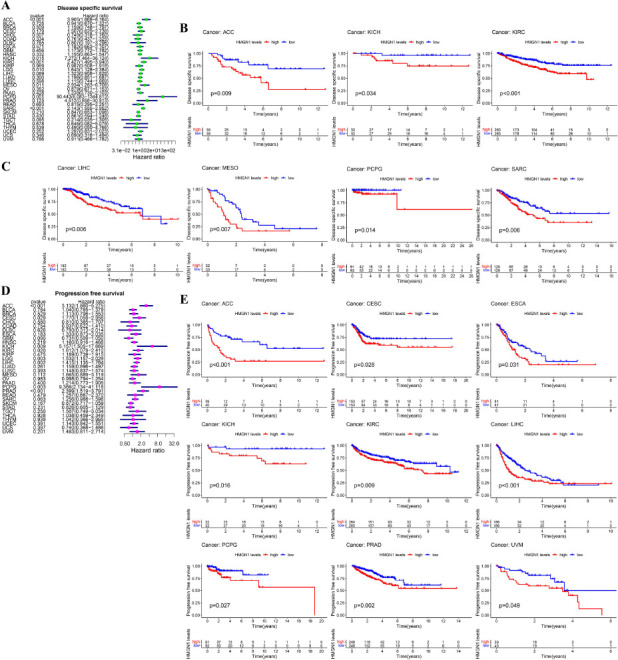
Association between HMGN1 expression and disease-specific survival (DSS) as well as progression-free survival (PFS). **(A)** Forest plot of DSS associations in 32 types of tumors. **(B, C)** Kaplan-Meier curve of the association between HMGN1 expression and DSS. **(D)** Forest plot of PFS associations in 32 types of tumors. **(E)** Kaplan-Meier curve of the association between HMGN1 expression and PFS.

Additionally, Cox analysis for DSS revealed that HMGN1 expression was inversely linked to the prognosis of ACC (*p<*0.001), LGG (*p*=0.010), KIRC (*p<*0.001), KICH (*p*=0.015), MESO (*p*=0.011), PCPG (*p*=0.001), and SARC (*p<*0.001) patients (Fig. **5A**). Meanwhile, K-M survival analysis (Figs. **5B**-**C**) indicated that elevated HMGN1 expression was linked to worse DSS results in ACC (*p*=0.009), KICH (*p*=0.034), KIRC (*p<*0.001), MESO (*p*=0.007), LIHC (*p*=0.006), PCPG (*p*=0.014), and SARC (*p*=0.006) patients. It was further noted that high HMGN1 expression levels were linked to poor PFS values in ACC (*p<*0.001), KICH (*p*=0.009), CESC (*p*=0.029), KIRC (*p*=0.020), LIHC (*p*=0.002), LGG (*p*=0.003), PCPG (*p*=0.003), and PRAD (*p<*0.001) patients (Fig. **5D**). The findings of the K-M analysis showed that patients with high HMGN1 expression levels in ACC (*p<*0.001), ESCA (*p*=0.031), CESC (*p*=0.028), KICH (*p*=0.016), KIRC (*p*=0.009), LIHC (*p<*0.001), PCPG (*p*=0.027), PRAD (*p*= 0.002), and UVM (*p*=0.049) patients presented a worse prognosis (Fig. **5E**).

**Fig. (6):**
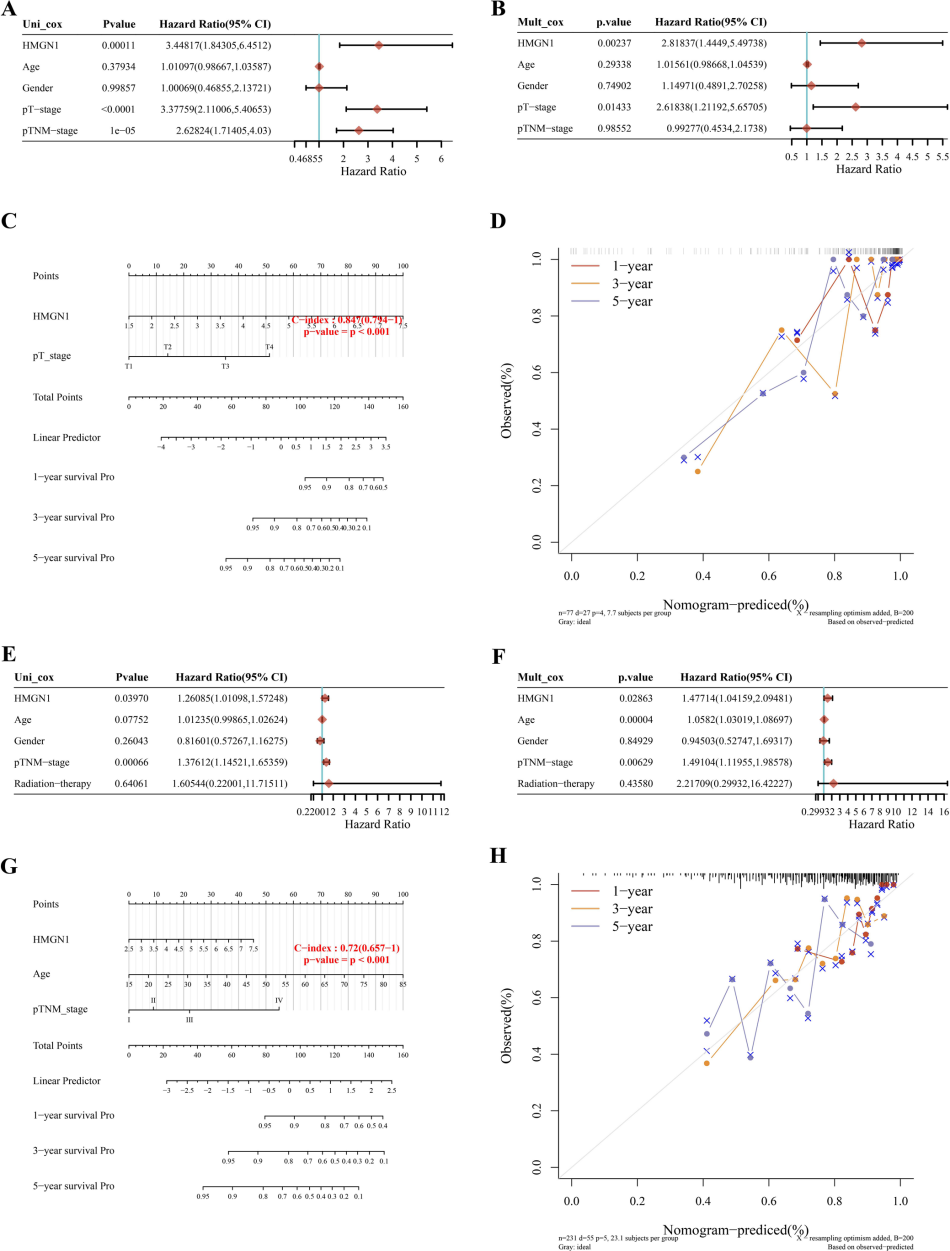
Establishment of the nomogram for prediction prognosis of ACC and liver hepatocellular carcinoma (LIHC). **(A, B)** Uni- and multivariate Cox regression analysis were conducted to uncover the association of clinical features and HMGN1 expression with the survival outcome of ACC. **(C)** A prognostic nomogram was exploited through integrating HMGN1 expression and pT_stage to estimate the survival probability of ACC. **(D)** The calibration plot of ACC. **(E, F)** Uni- and multivariate Cox regression analysis in LIHC. **(G)** A prognostic nomogram was exploited through integrating HMGN1, age, and pTNM_stage to estimate the survival probability of LIHC. **(H)** The calibration plot of LIHC.

Nomograms, which are frequently employed in cancer prediction, can precisely forecast the prognosis of specific patients by displaying them more graphically and visually. Since HMGN1 was linked to OS, DFS, DSS, and PFS in both LIHC and ACC, HMGN1 was integrated with clinicopathological variables in ACC and LIHC to develop predictive nomograms for cancer patients. HMGN1 was identified as the independent predictor of prognosis in ACC patients using the univariate and multivariate Cox regression models (Figs. **6A**-**B**). The data relating to HMGN1 expression and T-stage were used to construct the nomogram of ACC patients (Fig. **6C**). This nomogram displayed a C-index of 0.847. The findings of the calibration curve analysis indicated that 1-, 3-, and 5-year OS periods that were forecasted by the nomogram were closer to the OS duration (Fig. **6D**). A similar result was noted after analyzing the LIHC patients (Figs. **6E**-**H**).

### 3.5 Relationship between HMGN1 and the Immune Score and Stromal Score

**Fig. (7):**
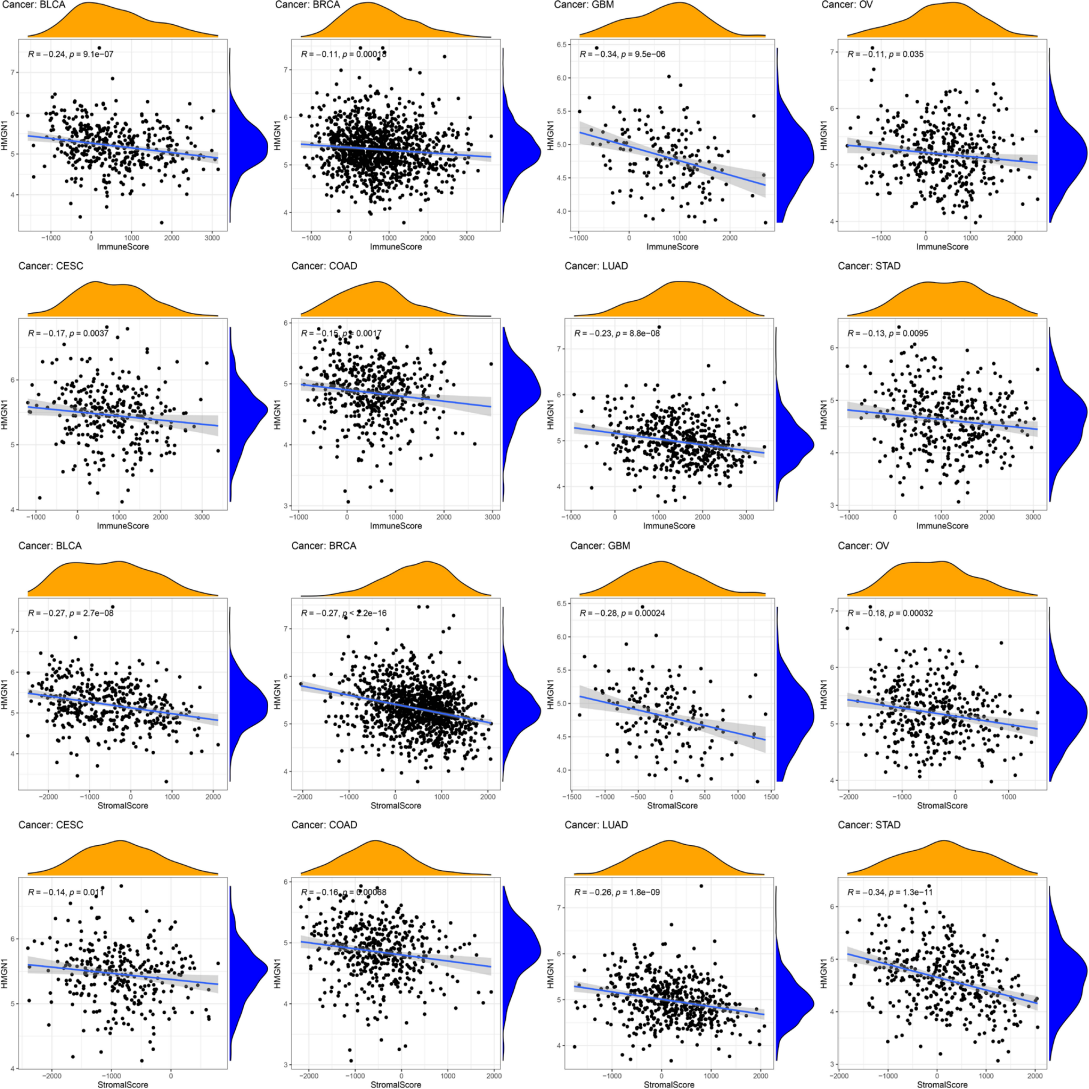
Correlation between HMGN1 and ImmuneScore and StromalScore in bladder urothelial carcinoma (BLCA), breast invasive carcinoma (BRCA), glioblastoma multiforme (GBM), ovarian serous cystadenocarcinoma (OV), cervical squamous cell carcinoma and endocervical adenocarcinoma (CESC), colon adenocarcinoma (COAD), lung adenocarcinoma (LUAD), stomach adenocarcinoma (STAD).

Several studies observed that TIME was significantly involved in tumor development. Therefore, the relationship between TIME and HMGN1 was assessed and the ESTIMATE algorithm was employed for assessing the stromal and immune scores in the malignant tumors. The findings revealed that stromal and immune scores showed a significant and negative link with the HMGN1 expression levels in eight types of cancers like BRCA, BLCA, COAD, CESC, GBM, LUAD, OV, and STAD (*p<* 0.05) (Fig. **7**).

### 3.6 HMGN1 Regulates the Tumor Infiltration of Immune Cells (TIICs) in Numerous Human Cancers

**Fig. (8):**
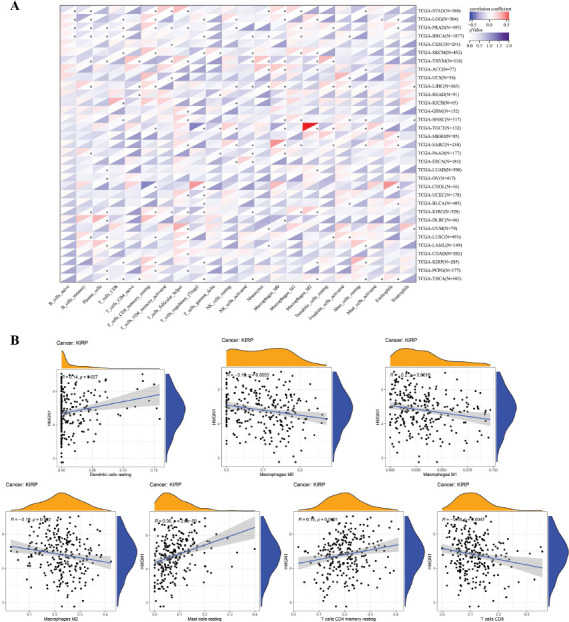
Correlation of HMGN1 expression with immune infiltration. **(A)** The heat map shows that HMGN1 expression correlated significantly with tumor infiltration of different immune cells from the CIBERSORT database. **(B)** Correlation of HMGN1 expression with infiltration of different immune cells in kidney renal papillary cell carcinoma (KIRP).

Furthermore, the correlation between the TIICs and HMGN1 levels was evaluated using the CIBERSORT algorithm. The findings of the heat map demonstrated that HMGN1 was significantly linked with a majority of TIICs (Fig. **8A**). It was also noted that the HMGN1 expression levels were positively linked to several TIICs in LGG and LIHC, while it was negatively linked to many TIICs in KIRC, BRCA, THYM, TGCT, and THCA. HMGN1 exhibited a significantly positive relationship with M2 macrophages in TGCT and a significantly negative relationship with Treg cells in TGCT. Additionally, the link between the HMGN1 expression in KIRP and different immune cell infiltration levels was also analyzed (Fig. **8B**). The findings demonstrated that the expression of HMGN1 was significantly and adversely correlated with M0, M1, M2, and CD8 T cells, but favorably correlated with resting CD4 memory T cells, resting mast cells, and resting dendritic cells.

**Fig. (9):**
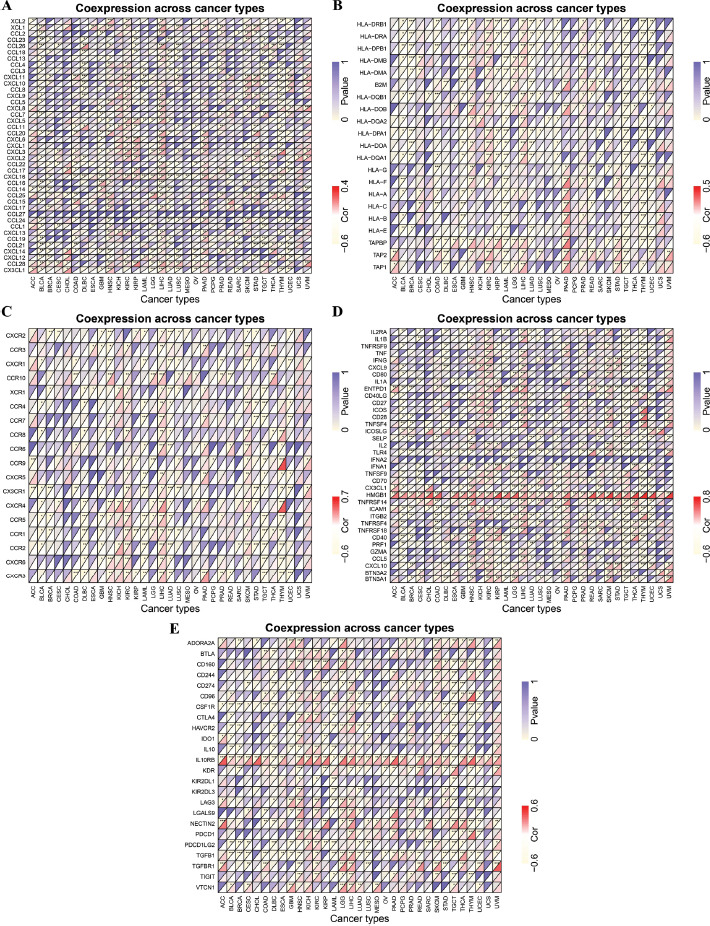
Co-expression of HMGN1 and immune-related genes in different cancers. The heatmap represented the correlation between HMGN1 expression and **(A)** Chemokines, **(B)** MHC genes, **(C)** Chemokine receptors, **(D)** Immune activation genes, **(E)** Immunosuppressive genes.

Additionally, gene co-expression analysis was employed to study the correlation between the genes associated with the immune system and HMGN1 expression levels in 33 cancers, such as chemokines (Fig. **9A**), MHC-encoding genes (Fig. **9B**), chemokine receptors (Fig. **9C**), immune-activating (Fig. **9D**), and immune-suppressing genes (Fig. **9E**). The heat map analysis revealed that a majority of the immune-linked genes were associated with HMGN1. The HMGN1 expression was significantly and positively linked with several immune-related genes in KIRC, HNSC, LIHC, and pancreatic adenocarcinoma (PAAD) cells. Additionally, few immune-related genes were found to be associated with HMGN1 in different cancers such as ACC, CHOL, ESCA, KICH, MESO, PCPG, and Uterine Carcinosarcoma (UCS).

### 3.7 Correlation between HMGN1 Expression Levels, TMB, MSI, and Immunotherapy Response

**Fig. (10):**
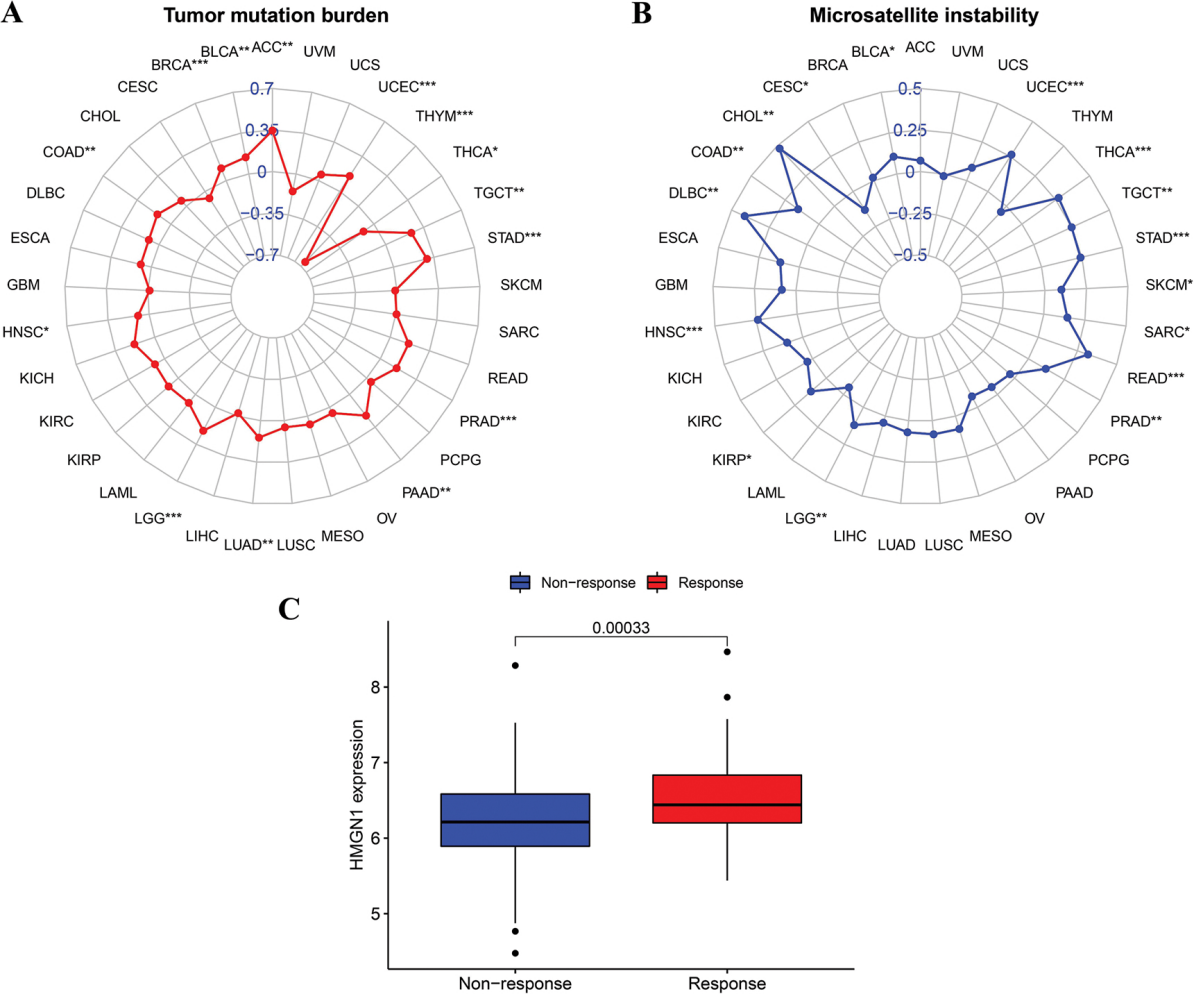
Associations between HMGN1 expression and tumor mutation burden (TMB), microsatellite instability (MSI), and immune response in different cancers. **(A, B)** Radar plots illustrating the relationship between HMGN1 expression and TMB as well as MSI. **(C)** Comparison of HMGN1 expression levels in immunotherapy responders and non-responders in the IMvigor210 cohort.

Antitumor immunity is linked to MSI and TMB and is a useful predictor that highlights the effectiveness of tumor immunotherapy. Immune checkpoint inhibitor therapy is more effective against tumors that display higher MSI and TMB values. As a result, the link between HMGN1 expression levels and MSI and TMB values (Figs. **10A**-**B**) was assessed to determine if HMGN1 was a predictor of immunotherapeutic response in different malignancies. The findings of this analysis revealed that HMGN1 expression was significantly and negatively linked with TMB in THYM and THCA, while it displayed a significantly positive relationship with TMB in different tumors such as BRCA, ACC, BLCA, COAD, LGG, HNSC, LUAD, PRAD, PAAD, STAD, TGCT, and UCEC (*p<*0.05). Also, HMGN1 expression was significantly and positively linked to MSI in CHOL, BLCA, HNSC, COAD, KIRP, Lymphoid Neoplasm Diffuse Large B-cell Lymphoma (DLBC), PRAD, LGG, Rectum adenocarcinoma (READ), SKCM, SARC, STAD, THCA, TGCT, and UCEC (*p<*0.05), but it showed a significant and negative relationship with CESC. Furthermore, when the HMGN1 expression levels in the IMvigor210 cohort between immunotherapy responders and non-responders (Fig. **10C**) were also compared, it was noted that the HMGN1 expression was significantly elevated in the immunotherapy responders (*p*=0.003).

### 3.8 The Analysis of GSEA

**Fig. (11):**
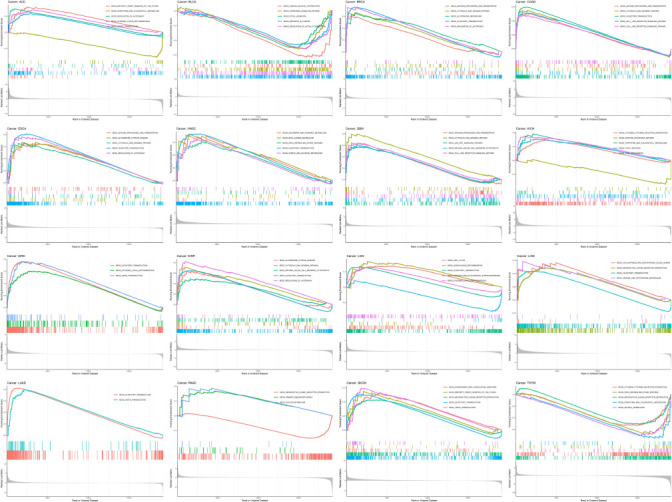
KEGG pathway analysis of HMGN1 in multiple cancers by GSEA. Curves of different colors showed different pathways regulated in different cancers. Peaks on the upward curve indicated positive regulation and peaks on the downward curve indicated negative regulation.

To further investigate its potential biological consequences, GSEA was used to assess the enrichment pathway of HMGN1 in 16 types of cancers (Fig. **11**). The results of GSEA revealed that HMGN1 influenced many KEGG pathways. For instance, three of the KEGG pathways—“CYTOSOLIC DNA SENSING PATHWAY,” “ANTIGEN PROCESSING AND PRESENTATION”, and “OLFACTORY TRANSDUCTION”—were simultaneously enriched in BRCA, COAD, ESCA, indicating that HMGN1 could exhibit a few common biological effects or employ similar mechanism in the above 3 types of cancers. Notably, HMGN1 was also seen to influence many metabolic pathways, such as “ASCORBATE AND ALDARATE METABOLISM”, “BETA ALANINE METABOLISM”, “PENTOSE PHOSPHATE PATHWAY”, “GLUTATHIONE METABOLISM”, “PENTOSE AND GLUCURONATE INTERCONVERSIONS”, “SULFUR METABOLISM”; while it also influences other immune-related pathways like “CYTOKINE CYTOKINE RECEPTOR INTERACTION”, “ANTIGEN PROCESSING AND PRESENTATION”, “NATURAL KILLER CELL MEDIATED CYTOTOXICITY”, “CHEMOKINE SIGNALING PATHWAY”, and “JAK-STAT SIGNALING PATHWAY”. The above results revealed suggested that HMGN1 assisted in the regulation of metabolism and immunity.

### 3.9 Drug Response of HMGN1

The GDSC database was then employed for assessing the link between HMGN1 expression levels and drug sensitivity of various chemotherapeutic drugs. As demonstrated in Fig. (**12**), HMGN1 expression levels were positively linked to the IC_50_ values of chemotherapeutic drugs such as Amonafide, 3-Bromopyruvate, Chelerythrine, Dexrazoxane, Ifosfamide, Pyrazoloacridine, Nelarabine, LMP776, Palbociclib, PX-316 Oxaliplatin, Parthenolide, and 8-Chloro-adenosine, while HMGN1 expression levels were negatively linked to the IC_50_ values of Elliptinium Acetate, Vemurafenib, and Mithramycin.

### 3.10 Validation of HMGN1 Expression

**Fig. (13):**
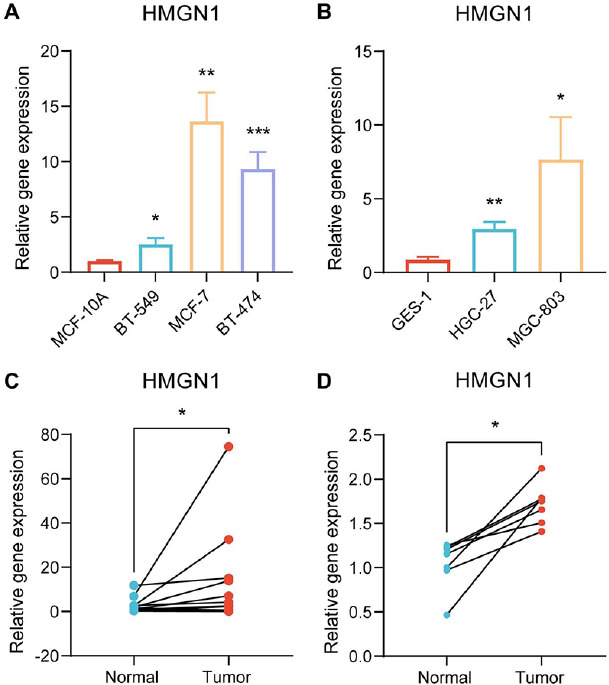
Validation of HMGN1 expression. **(A)** qRT-PCR analysis of HMGN1 in breast cancer cell lines and normal cell line MCF-10A. **(B)** qRT-PCR analysis in stomach cancer cell lines and normal cell line GES-1. qRT-PCR analysis of HMGN1 in breast cancer **(C)** and stomach cancer **(D)** tissues.

Finally, immunohistochemistry and PCR techniques were employed to verify the HMGN1 expression levels in the malignant and healthy tissues. HMGN1 protein was significantly over-expressed in five cancer tissues, such as breast cancer, liver cancer, lung cancer, prostate cancer, and colorectal cancer, compared to the healthy tissues, as shown in Supplementary Fig. (**1**). Furthermore, the findings of the PCR experiments indicated that HMGN1 was over-expressed in the gastric and breast cancer cell lines in comparison to the healthy cell lines (Figs. **13A**-**B**). It was also noted that the HMGN1 expression was elevated in the gastric and breast cancer samples compared to the paraneoplastic tissues (Figs. **13C**-**D**).

## 4 DISCUSSION

Here, we examined the involvement of the HMGN1 gene in cancer prediction, progression, and treatment by thoroughly analyzing the HMGN1 gene molecular signature in 33 types of cancers, derived from multiple portals such as the TCGA, GTEx, and UALCAN.

HMGN1 is a highly conserved chromatin structural protein that regulates histone modification, chromatin structure, and gene expression and participates in a variety of biological activities [25]. In some cases, the HMGN1-based chromatin remodeling regulation can result in transcriptional repression [12, 26, 27], while in other instances, it supports transcriptional activation [27-30]. It should be noted that HMGN1 inhibits the growth of malignant cells. The findings further indicated that the HMGN1 gene binds to the promoter of the proto-oncogene FosB and inhibits histone H3 phosphorylation on H3S10P, to suppress its expression [31]. Additionally, it was noted that HMGN1 -/- mice developed 2-times the number of spontaneous tumors than their HMGN1 +/+ littermates [14]. However, paradoxically, Zhiheng *et al.* noted that HMGN1 was over-expressed in the malignant gastric tissues compared to the healthy gastric tissues [19]. In another study, Feng *et al.* observed that the serum HMGN1 levels were elevated significantly in the malignant non-small cell lung samples in comparison to the healthy and normal control samples, indicating that they were positively related to the tumor stages [32]. It was noted that the patients displaying high HMGN1 levels exhibited low OS levels following radical pneumonectomy compared to patients with lower serum HMGN1 levels. HMGN1 has also been identified as a target of recurrent DNA copy increase in leukemia, and the mechanisms that combined HMGN1 overexpression and the AML-ETO9a fusion oncoprotein could hinder bone marrow differentiation and promote leukemic stem cell activity [33]. This discrepancy could be attributed to the complicated involvement of HMGN1 in the bidirectional regulation of chromatin remodeling. Based on the data derived from the TCGA and GTEx databases, our findings implied that HMGN1 was significantly overexpressed in 26 types of cancers in comparison to the normal tissues. Furthermore, data from the UALCAN database demonstrated that the HMGN1 protein was over-expressed in the malignant colon, breast, glioblastoma multiforme, LIHC, LUAD, and ovarian cancer tissues compared to the healthy tissue samples. Furthermore, it was also noted that high HMGN1 expression levels were linked to the late clinical stages in ACC, KICH, and KIRC. Additionally, numerous survival analyses were conducted and the findings showed that high HMGN1 expression levels were responsible for poor prognosis of ACC, ESCA, CESC, KIRC, KICH, LIHC, LGG, PCPG, SARC, MESO, PRAD, and UVM patients. The results have highlighted the role played by HMGN1 as a probable prognostic biomarker.

The TIME, which is made up of immune cells, stromal cells, and tumor cells, is crucial for the growth, recurrence, and drug resistance of malignant cells [34-37]. The TIME properties can be employed as markers for assessing the tumor cell immunotherapy response and are seen to affect the therapeutic outcomes. According to ESTIMATE scores, the stromal and immune cell concentrations were inversely related to the HMGN1 expression in the TIME of 8 types of cancers. TIICs are a vital component of TIME and thus are linked to the growth, infiltration, and metastasis of cancer cells [38, 39]. The results presented in an earlier study showed that HMGN1 interacts with Gαi protein-coupled receptors to promote dendritic cell recruitment [40]. Based on the findings of all experiments, we noted that HMGN1 is strongly associated with several immune cells. Furthermore, it was seen that HMGN1 was associated with immune-linked genes that code for the MHC, immune activation, immunosuppression, chemokines, and chemokine receptors. MSI and TMB are regarded as favorable predictive biomarkers for prognosis following immunotherapy in pan-cancer patients [41-44]. The findings of the correlation study between HMGN1 levels and TMB/MSI revealed that TMB was linked to HMGN1 expression in 14 cancers, while MSI was associated with HMGN1 expression in 16 types of cancers. Additionally, numerous studies have demonstrated that HMGN1 enhances anticancer immunity by reversing T-cell depletion and increasing the response of malignant cells to cancer vaccines [45-47]. The HMGN1 expression was considerably higher in the immunotherapy responders in the IMvigor210 group, which was in line with earlier findings indicating that HMGN1 supports antitumor immunity. These findings collectively imply that HMGN1 may be an effective immunotherapy target, which offers hope for the clinical management of tumor patients.

Furthermore, our enrichment analysis reveals that HMGN1 may be crucial in controlling immunological and metabolic pathways. The results presented here are in agreement with the earlier studies that claimed the HMGN1 deletion leads to histone inactivation, modification of the extracellular proteome, and immune regulatory pathways [48]. The use of computational models and public databases to discover patient-sensitive drugs is becoming increasingly common [49, 50]. Finally, the GDSC database was employed for assessing the connection between HMGN1 expression levels and drug sensitivity of various medications for different cancers, offering a theoretical foundation for clinical use.

However, this study has several drawbacks. The primary focus of our research lies in the analysis of transcriptomes. Our investigation of HMGN1 protein is limited to the examination of data obtained from the UALCAN and HPA databases. It is necessary to incorporate additional proteomic data into our analysis. Furthermore, the findings indicate that HMGN1 is connected to the invasion of immune cells in several tumors, however, the exact mechanism is yet unknown. Further research is warranted to validate the presence of HMGN1 expression in supplementary tumor samples and establish a connection between HMGN1 levels and the survival rates of cancer patients.

## CONCLUSION

In this study, an integrative analysis was conducted to investigate the expression patterns, prognostic value, and relevant immune and pharmacogenomic features of HMGN1. The findings revealed that HMGN1 exhibits elevated expression levels in diverse cancer types, indicating its potential as a promising biomarker for predicting prognosis. Additionally, a correlation was observed between HMGN1 and immune cell invasion, suggesting the potential utility of HMGN1 in immunotherapy and providing novel avenues for future research.

## AUTHORS' CONTRIBUTIONS

Y.G.: Conceptualization, Writing - original draft, Funding acquisition. R.Z.: Validation, Writing - original draft. H.X.: Validation, Writing - original draft. K.H.: Investigation. K.C.: Methodology, Software. Y.M.: Investigation. Z.W.: Writing - review & editing, Project administration.

## Figures and Tables

**Fig. (3):**
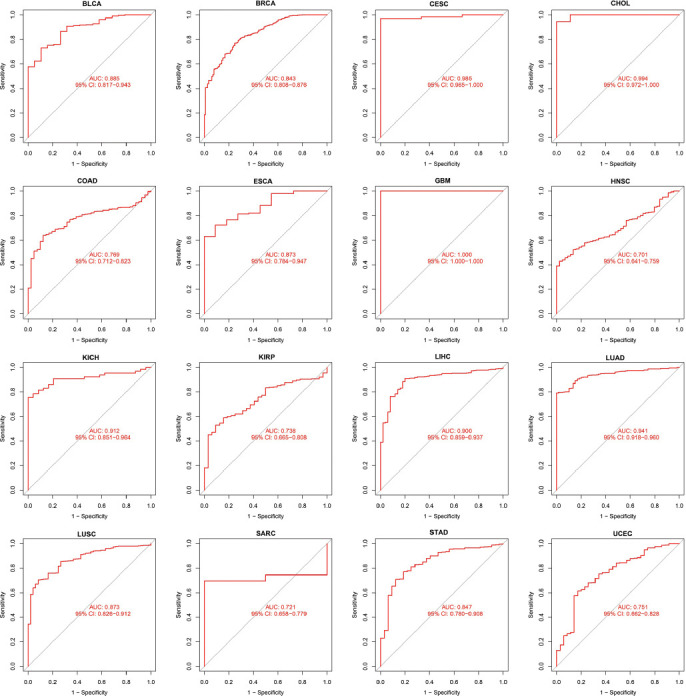
ROC analysis of HMGN1 genes in TCGA database.

**Fig. (12):**
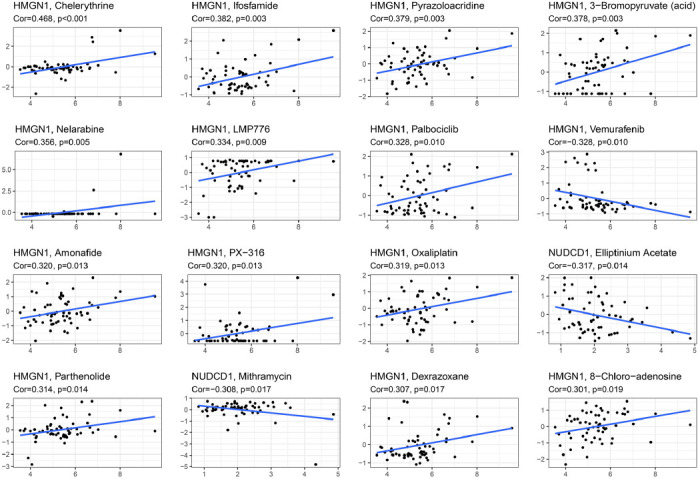
Correlation of HMGN1 expression with drug sensitivity in GDSC database.

## Data Availability

The datasets supporting the conclusions of this article are included within the article.

## LIST OF ABBREVIATIONS

UCS: Uterine Carcinosarcoma
TIICs: Tumor Infiltration of Immune Cells
HR: Hazard Ratio
MESO: Mesothelioma
PCPG: Pheochromocytoma and Paraganglioma
UCEC: Uterine Corpus Endometrial Carcinoma

## STANDARDS OF REPORTING

STROBE guidelines were followed.
