# Supplementary material for: Pan-cancer Multi-omics Analysis Reveals HMGN1 as a Potential Prognostic and Immune Infiltration-associated Biomarker
Source: Curr Med Chem. 2024 Jan 8;32(12):2440–59. doi: 10.2174/0109298673268718231122103638 (PMC12307959; doi:10.2174/0109298673268718231122103638)
Supplement: Supplementary file 1 [file CMC-32-12-2440_SD1.pdf]

Supplementary Material

Pan-cancer Multi-omics Analysis Reveals HMGN1 as a Potential Prognostic and Immune Infiltration-associated Biomarker

Yangyang Guo[1,#], Rongrong Zhang[2,#], Hongjie Xu[2,#], Kai Hong[1], Kenan Cen[1], Yifeng Mai[1] and Zhixuan Wu[2,*]

[1]Department of Thyroid and Breast Surgery, The First Affiliated Hospital of Ningbo University, Ningbo, 315010, Zhejiang, People's Republic of China; [2]Department of Pathology, The First Affiliated Hospital of Wenzhou Medical University, Wenzhou, 325000, Zhejiang, People's Republic of China

Table S1.

Human HMGN1	FORWARD	TGAAGCAGGAGAGAAAGAAGCCAAG
Human HMGN1	REVERSE	ACAAGAAGGGAGACAGGGACCAC

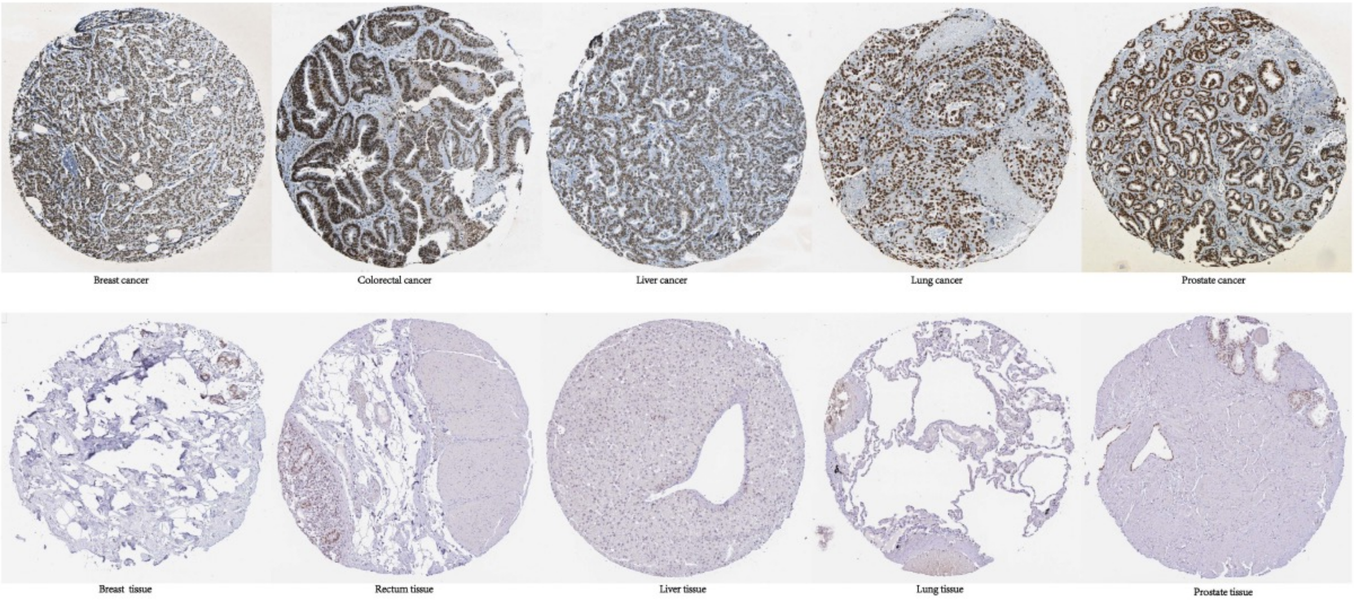

Fig. (S1). Immunohistochemical stainings of HMGN1 in cancers were procured from the Human Protein Atlas (HPA) (http://www.proteinatlas.org/) database. We detected the HMGN1 protein expressions in breast cancer, colorectal cancer, liver cancer, lung cancer, and prostate cancer.
